# Opposite effects of interruption frequency on performance in interrupted and uninterrupted multistep task

**DOI:** 10.1007/s00426-026-02270-0

**Published:** 2026-03-25

**Authors:** Tara Radović, Patricia Hirsch, Iring Koch, Torsten Schubert

**Affiliations:** 1 Institute of Psychology, Martin Luther University of Halle-Wittenberg, Emil-Abderhalden-Str 26-27, 06108 Halle, Germany; 2 Institute of Psychology, RWTH Aachen University, Jaegerstrasse 17-19, 52066 Aachen, Germany

## Abstract

We investigated the effects of interruption frequency on interrupted and uninterrupted performance in a serial multistep task. The multistep task consisted of five steps, which were memorized and executed consecutively in predefined order. This task was occasionally interrupted by a letter classification task. After completion of that interruption task, participants needed to resume the multistep task at the correct step. We measured response times and sequence errors (deviations from the prescribed order of steps) at the step after an interruption (resumption performance) and in the corresponding steps of uninterrupted tasks. Interruption frequency varied between subjects and was manipulated twofold as: (1) the percentage of interrupted tasks at all (global frequency: 25% vs. 75%); (2) the number of interruptions per task (local frequency: one or three interruptions), resulting in four different groups. The results revealed faster and more accurate resumption in both 75%-frequency groups compared to the lowest frequency group. However, participants in the highest-frequency group were slower in uninterrupted tasks compared to all other lower frequency groups. These results show that high interruption frequency in multistep tasks induces strategic shifts in participants’ control mode causing better performance during interrupted tasks at the cost of worse performance in uninterrupted tasks.

## Introduction

Interruptions of the ongoing activity are an unavoidable aspect of everyday life. This relates to situations at a workplace as well as to leisure and other situations. For example, at the workplace, we might be writing a report, when a colleague drops by for a chat. Afterwards, we need to resume the interrupted task, writing the report, where we left off. While such situations might take some additional time to re-orient to the interrupted task, in some other working environments such interruptions are not only prevalent but also pose a risk of committing an error. For example, in very dynamic working fields, such as intensive care units or emergency medicine, medical staff gets interrupted up to 23 times per hour (e.g., Drews et al., 2019; Grundgeiger & Sanderson, 2009; Ratwani et al., 2016). Due to their prevalence and the potential security risk they bear, there is a growing body of research investigating the performance consequences of interruptions from the perspectives of applied psychology as well as cognitive psychology to understand the underlying mechanisms.

Previous research provided fairly consistent evidence that the occurrence of interruptions has serious detrimental effects on the performance in interrupted tasks, that is, the primary tasks. As one possible consequence, interruptions (i.e., secondary tasks) often lead to forgetting to resume the primary task after an interruption completely (e.g., Einstein et al. 2003; Dodhia and Dismukes 2009; Grundgeiger et al. 2010a; see also Couffe & Michael, 2017; Hirsch et al., 2023, for review). Besides that, if the intention of resuming the primary task after the interruption is not completely forgotten, the resumption process comes at a cost and often leads to increased response times and increased probability of committing an error upon resumption (e.g., Altmann et al. 2014; Grundgeiger et al. 2010b; Hodgetts and Jones 2006; Radović and Manzey 2019, 2022).

Previous research focused usually on the effects of single interruptions on the performance in the primary task (e.g., Hirsch et al., 2024; Piątkowski et al., 2024), although interruptions typically occur not only once but even several times during a single primary task. Thus, whether and how the interruption effects are modulated by interruption frequencies largely remains an open issue up to now. Some of the previous studies have addressed this question, but results seem inconsistent in this regard. On one hand, several studies (e.g., Basoglu et al. 2009; Lee and Duffy 2015; Speier et al. 1999; Westbrook et al. 2010a, b) provided evidence that frequent task interruptions increase resumption times and error rates after the interruption compared to a condition with less frequent task interruptions. For example, in a study of Speier et al. (1999), problem-solving tasks (e.g., to rank warehouses based on cost estimates and to determine the number of goods to produce based on forecasts) were interrupted either four times (low-frequency condition) or 12 times per problem-solving task (high-frequency condition). The results revealed that participants needed overall more time to solve a problem and made more errors in the high-frequency condition compared to the low-frequency condition. Similarly, in an observational study of Westbrook and colleagues (2010b) the probability of committing a medication error increased with each additional interruption. On the other hand, the findings of other studies seem to be in contrast to that (e.g., Monk, 2004; Powers & Scerbo, 2022; Radović et al., 2022). In more detail, Monk (2004) found significantly faster and even more accurate performance after an interruption when interruptions occurred with a high frequency (every 10s) compared to a low frequency (every 30s). Also, in a study by Powers and Scerbo (2022), shorter resumption times were found in a decision-making primary task (planning a weekend) in the high-frequency condition (6 interruptions per planning task) compared to the low-frequency condition (2 interruptions).

The inconsistent findings in previous studies might be caused by different reasons. For example, different operational definitions of interruption frequency might have contributed to the observed inconsistent pattern of findings on this issue. Specifically, in some studies, interruption frequency was varied locally on the level of a primary task, that is, three or 12 interruptions per one problem-solving task (e.g., Speier et al., 1999). However, in other studies, interruption frequency was manipulated more globally on the experiment level, that is, by interrupting 25% vs.75% of all tasks in the experiment only once (e.g., Radović et al., 2022). Finally, some studies did not specify at all on which level the interruption frequency was manipulated (e.g., Westbrook et al. 2010b; Zijlstra et al. 1999). Thus, it is possible that different types of interruption frequency manipulations, which target either the level of a single primary task or the processing of the task on the experiment level, can have different effects on the performance in the primary task. On one hand, interrupting one primary task for several consecutive times might have negative effects on task performance, because this might impair the formation of an appropriate task strategy. On the other hand, interrupting one primary task only once but with a higher frequency across the whole experiment might even have a positive effect on performance in the primary task, perhaps due to accumulating experience with interruption processing or other reasons. Indeed, there is evidence suggesting that more experience with interruptions facilitates the resumption of a primary task (e.g., Cades et al., 2011; see also Altmann & Hambrick, 2017, for negative effects of practice on sequence errors). This evidence comes from studies in which participants practiced to handle task-interruption demands.

Besides the different types of manipulations of interruption frequency, previous studies often focused on different aspects of performance in the primary task. Thus, some studies focused on general performance measures, such as the total time spent on the primary task (e.g., Speier et al., 1999), the total number of errors (e.g., Westbrook et al. 2010a), or some more complex derivative of these two measures (e.g., Lee & Duffy, 2015). At the same time, other studies analyzed rather specific aspects of performance, such as the time needed to complete the first step of a multistep task after an interruption (resumption time; e.g., Monk, 2004; Powers & Scerbo, 2022). Therefore, it is conceivable that different types of interruption frequency manipulations might affect different aspects of performance such as the resumption efficiency in the interrupted primary task, the processing of the primary task by itself (i.e., uninterrupted primary task), or other aspects in a different way. For example, higher interruption frequency might have a positive effect on the time needed to resume the primary task at the first step directly after an interruption. However, at the same time, it could also lead to a negative effect on the error rates or to a negative effect on a more general level, for example, on the performance in uninterrupted primary tasks.

Considering the above-mentioned conceptual and methodological differences between studies, the present study aimed to investigate how interruption frequency impacts performance in a primary task that consists of several task steps which have to be executed in a predefined order, that is, a serial multistep task (e.g., Altmann et al., 2014; Kopacz et al., 2019; Radović & Manzey, 2019). Generally, this type of primary task is interesting for investigating the effects of interruptions because response times at each step of the task can be measured, as well as two different types of errors - sequence errors (repeating or skipping a step in the task) and non-sequence errors (wrong choice of the response alternative at one step; see also Moretti et al., 2024). Interestingly, previous studies using this type of primary task found larger interruption costs specifically in terms of response times and sequence errors when resuming the primary task after an interruption, while non-sequence errors remain unaffected (e.g., Altmann et al., 2014; Kopacz et al., 2019; Monk et al., 2008; Radović & Manzey, 2019). Thus, the negative effects of interruptions are attributed to an additional effort related specifically to the re-activation of goals of the primary task, and not to a general disruption of attentional or cognitive resources. Moreover, and specifically relevant in the context of the present study, this particular type of primary task is suitable for investigating the impact of interruption frequency on different levels of the task and different aspects of task performance. In more detail, it allows us to investigate the interruption effects on the local task level by comparing interruption effects between situations in which the multistep task is interrupted only once or several times between different steps (the latter would not be possible in a single-step task). In addition, it allows us to investigate interruption effects on a more global level by analyzing the potential influence of the total number of multistep tasks that get interrupted at all during the whole experiment (independently of whether the task is interrupted once or several times). Therefore, we investigated the effect of interruption frequency on the performance in the primary task separately for the interrupted tasks, which reflects a measure of resumption performance (i.e., at the first step after an interruption), and in uninterrupted multistep task. In this case, one task is defined as a complete pass through all steps of the multistep task. This particular design of manipulating the interruption frequency allows us further to relate different theoretical positions for deriving predictions about the effects of interruption frequency on performance in interrupted tasks and uninterrupted tasks.

According to Memory for Goals (MfG) theory (Altmann & Trafton, 2002) the processing of task interruptions can be understood by considering the goal representation of the tasks in working memory. The theory assumes that task goals maintained in memory have different activation levels and the task goal with the highest activation level will guide the ongoing behavior. To increase the activation level of a relevant task goal above the level of other irrelevant goals (i.e., interference level), a mechanism of strengthening is employed. During the strengthening process, the system encodes the task goal and samples the goal repeatedly, which increases its activation level. The strengthening process operates cumulatively, meaning that each sampling of the goal accumulates on previous retrievals. Importantly, if a goal is not relevant anymore, which is the case while performing the interruption task, the activation of the primary task will decay with time. After the interruption has finished and the primary task has to be resumed, re-activation of the goal of the primary task will take place. Re-activation of former goals can be supported by a priming mechanism using internal or external cues related to the target goal. This re-activation process is time-consuming and error-prone, which is reflected in higher response times and increased error rates when resuming the primary task after an interruption compared to the performance in uninterrupted tasks. Moreover, goals have a base-level activation which is determined by the goal’s history of use with more frequently and recently retrieved goals having a higher base-level activation than other goals. According to these assumptions, one would assume that if the primary task is interrupted more often, the goals of the primary task also have to be re-activated more often in order to resume this task after an interruption. The frequent retrieval should have a cumulative impact on the activation level of the goals in memory, which would make them more accessible for recall. If this were the case, we should expect a general improvement of the performance in the primary task, which can be observed in better performance on interrupted tasks requiring resumption and on the uninterrupted primary tasks because in both situations increased task activation would be beneficial.

However, a different view on the potential effects on interruption frequency on the interrupted and the uninterrupted task situations becomes possible when additionally considering the impact of interruption frequency on participants’ task strategies and control modes (e.g., Braver, 2012; Braver & Barch, 2002). According to the Dual Mechanism of Control (DMC) framework of Braver (2012), there are different modes of cognitive control that differ in their temporal dynamics, namely, a proactive mode and a reactive mode of cognitive control. The proactive mode of cognitive control is assumed to result in preparation before a task in which the goal-relevant information is ready and actively maintained before the occurrence of a critical task. This supports anticipation and prevention of interference before it occurs, by optimally biasing attention, perception, and action according to the goals. On the other hand, the reactive mode of cognitive control is considered to be a late-correction mechanism, which is activated after an interfering event had been detected. Several studies could show that the frequency with which an interfering event would occur influences the degree to which each of these control modes is recruited during task processing (Bonnin et al., 2011; Dreisbach & Haider, 2006; Strivens et al., 2024). For example, in their study on the effects of frequency on task-switching performance, Dreisbach and Haider (2006) found that in high-demand blocks with a high frequency of task switching (75% of trials), participants showed faster responses on switch trials and slower responses on repetition trials. These effects can be explained by dynamic preparation that is adjusted to the expected task demands specifically (i.e., switching). By analogy with these findings, one would expect that in interrupted tasks a high interruption frequency would increase the expectancy of interruptions and, thus, increase the activation of a proactive mode of cognitive control to deal with the potential behavioral consequences of the interruption. Such strategic adjustments can be of help to manage the mechanisms to control and to deal with the occurring interfering information during interruption in a more effective way, which can lead to improved performance when the expected interference is occurring, that is, specifically for the interrupted tasks. Such preparation involves mechanisms such as more efficient encoding or retrieval of the most recently completed goal of the interrupted primary task, reconstruction of its task space, or retrieval of the currently relevant task goal at the point of resumption. As a consequence, this preparation should lead to faster and more accurate resumption performance in the interrupted tasks. At the same time, a shift to a more proactive control mode associated with a higher expectancy of interruptions should be detrimental to the processing of the uninterrupted tasks because the system has adjusted the control mode for an interrupted but not uninterrupted processing. In other words, such preparation for dealing with frequent interruptions should not be useful in the rare situations in which the interruption is not present. This dynamic of the control modes would suggest the rather paradoxical prediction that when the uninterrupted task is processed in a situation with highly frequent interruptions, the performance in the uninterrupted tasks will suffer and result in higher response times and a higher error rate compared to a low interference condition.

In sum, the rationale outlined above results in the following set of hypotheses that enable us to distinguish between the two theoretical frameworks for understanding interruption frequency effects for performance, which is the primary aim of the current research. Critically, the two theories make differential predictions regarding the performance in the interrupted and uninterrupted tasks in relation to the overall number of interruptions. While the MfG framework predicts the same tendency of the impact of different interruption frequencies for both types of tasks, the DMC approach predicts an opposite dynamic between the two types of tasks. Thus, in order to distinguish between the two theories, the following hypotheses on the effects of interruption frequency refer to the overall number of interruptions. In addition, it would be of further interest to specify whether effects of interruption frequency vary depending on the structure of the task, that is, at which level of the task these interruptions take place. For example, it is an open question whether it makes a difference if the interruption is occurring several times within one multistep task or only once. These differences will be addressed by including the interruptions located at the local level (i.e., number of interruptions within one multistep task) and at the global level (i.e., number of multistep tasks interrupted at all). However, because the MfG and DMC theories do not make differential hypotheses about the level at which interruptions occur, these issues will be investigated as subordinate goals in the current investigation and will be addressed by additional analyses in an exploratory manner.

According to the MfG theory (Altmann & Trafton, 2002) we would expect that increasing interruption frequency would be accompanied by faster and more accurate performance in terms of sequence errors in both the interrupted and uninterrupted multistep primary tasks. This hypothesis is based on the assumption that an increase in interruption frequency leads to a more frequent sampling of the goal of the primary task which would consequently increase the activation level of the task-goal representation in memory and improve its accessibility for retrieval.

The DMC framework (e.g., Braver, 2012) predicts for the case of interrupted tasks that resumption performance should improve (i.e., faster responses and fewer sequence errors) in situations with higher compared to situations with lower interruption frequency. This would be in accord with the assumption that increasing interruption frequency would be accompanied by a strategic shift of participants’ control mode toward a better preparation of the system for interruption processing. However, such a shift of the control to a proactive mode (i.e., preparation for interruption) should be accompanied by detrimental effects on the performance in the uninterrupted tasks, which would be reflected in slower responses and more sequence errors in this type of task as the interruption frequency increases.

The aim of the present study was to test these predictions with a task situation in which participants performed a serial multistep task which was occasionally interrupted by a letter categorization task. We administered four interruption frequency conditions to separate groups of participants. The overall interruption frequency for each group resulted from the number of interruptions within one multistep primary task (i.e., either one or three interruptions per primary task; the local level) multiplied by the number of interrupted primary tasks at all (i.e., either 25% or 75% of all primary tasks interrupted at all; the global level). This resulted in the following combination of interruption frequency groups: the low frequency group with 24 interruptions (i.e., low frequency at both the local and global levels), two middle frequency groups with 72 interruptions each (i.e., one group with a high frequency at the local level but a low frequency at the global level and the other group with a low frequency at the local level but a high frequency at the global level), and the high frequency group with 214 interruptions in total (i.e., high frequency at both the local and global levels).

## Method

### Tasks

**Fig. 1:**
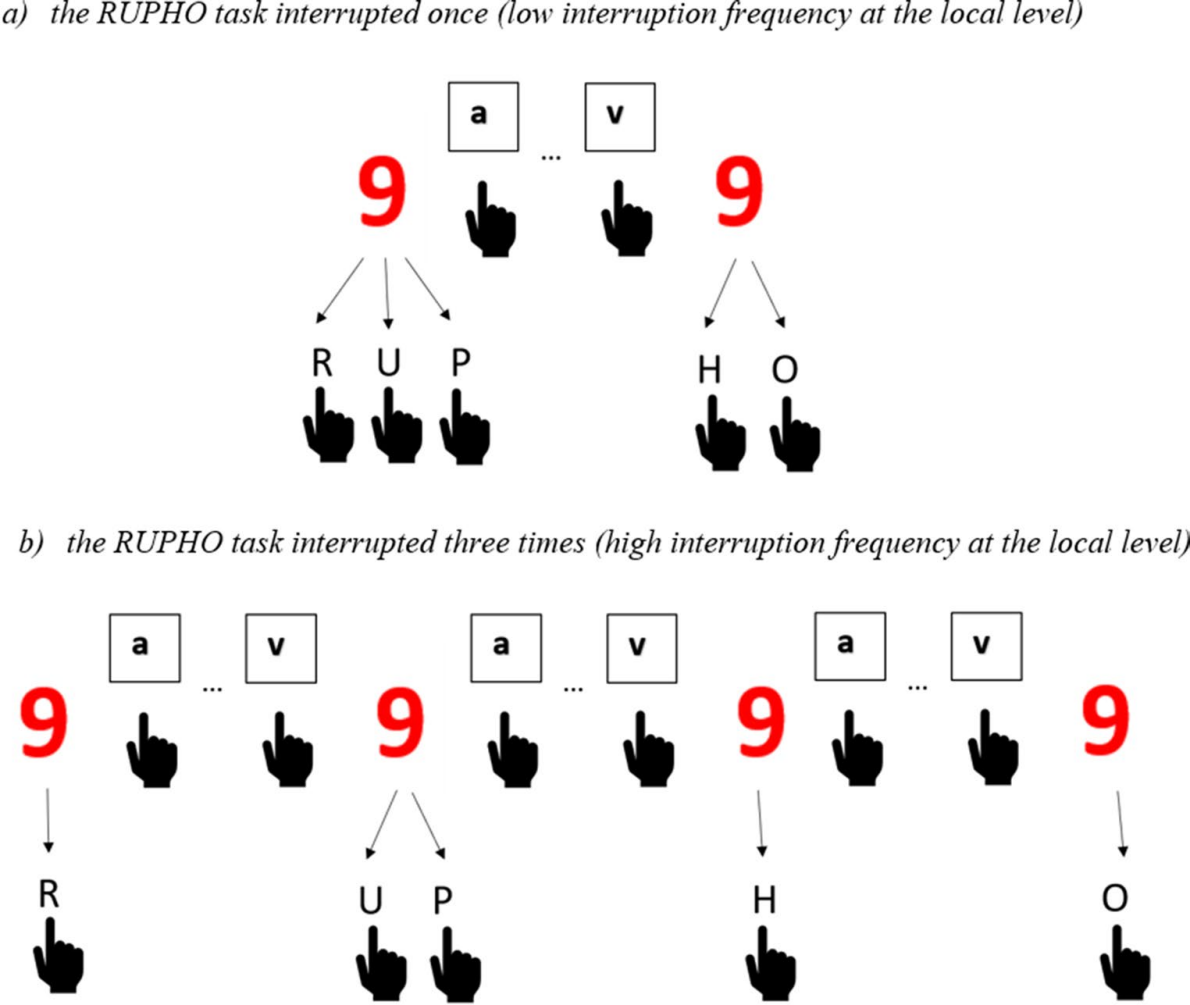
Schematic depiction of one task

Primary task. We administered a novel multistep task, the RUPHO task, as the primary task (for similar tasks see Altmann et al., 2014; Hirsch et al., 2023; Kopacz et al., 2019; Radović & Manzey, 2019). This primary task consisted of five consecutive steps, which had to be learned and then executed in a predefined order (see Table 1). At each step, participants had to evaluate one out of five different properties of a digit (red or blue; odd or even; central or peripheral to 5^1^; below or above 5; with or without a line) in consecutive order and respond by pressing a specifically assigned key on a standard keyboard. Each step was linked to a unique pair of response keys. A set of possible key responses is presented in Table 1. As it can be seen in Table 1, one set of possible response keys forms a mnemonic acronym RUPHO. One multistep primary task was bound to only one-digit stimulus and required all five properties to be evaluated one after the other (either with or without interruption). Combining the five properties with eight possible digits (1, 2, 3, 4, 6, 7, 8, 9) allowed for 32 unique digit stimuli. An example of the stimuli can be seen in Fig. 1.

**Table 1:** RUPHO task: choice rules, alternatives and response keys. English translation of the choice rules and alternatives is included in brackets

	Choice rule	Alternatives	Response keys
R	Ist die Zahl rot oder blau?(*engl*. Is the number red or blue?)	rot, blau(red, blue)	R, B
U	Ist die Zahl ungerade oder gerade?(*engl*. Is the number odd or even?)	ungerade, gerade(odd, even)	U, G
P	Ist die Zahl peripher oder zentral zu 5? (*engl*. Is the number peripheral or central?)	peripher, zentral(peripheral or central)	P, Z
H	Ist die Zahl höher oder niedriger als 5?(*engl*. Is the number higher or lower than 5?)	höher, niedriger(higher, lower)	H, N
O	Ist die Zahl ohne oder mit Strich?(*engl*. Is the number without or with line?)	ohne Strich, mit Strich(without, with)	O, M

Interruption task. The interruption task was a letter classification task. Participants were presented with a single letter (from the set: a, e, i, f, v, x), one at a time in the center of the computer screen, and had to classify them as a consonant by pressing the *1* key or as a vowel by pressing the *0* key on the keyboard using their index fingers. In total, 10 letters were presented in the interruption phase and participants completed the task in a self-paced manner.

### Apparatus and stimuli

Participants performed the primary task with occasional interruptions from a letter classification task on a BENQ screen (1920 × 1200 px, sampling with 60 Hz). Stimulus presentation and response recording were controlled by a Psychopy version 2022.2.5 (Peirce et al., 2019). All stimuli were presented centrally on a computer screen with a visual angle of 52° x 0.31° at a viewing distance of 80 cm. In the primary RUPHO task, digit stimuli (size 1.5 × 2 cm) were presented against a white background (Psychopy RGB (1, 1, 1). In the interruption task, participants were presented with a single letter (size 1 × 1 cm, lower case) in black color (Psychopy RGB (0, 0, 0) against a white background (Psychopy RGB (-1, -1, -1).

### Procedure

Ethical approval for this study was obtained from the ethics committee of Institute für Psychologie und Arbeitswissenschaften (IPA), Technische Universität Berlin, as a part of the DFG project MA 3759/5 − 1. Participants were tested individually in a single 90-minute experimental session at Martin Luther University of Halle-Wittenberg. Upon arriving at the lab, participants were introduced to the study and signed an informed consent, and filled out a demographic questionnaire (age, gender, self-assessment of their typing proficiency). Afterward, participants were randomly assigned to one of the four groups.

All groups got introduced to the primary RUPHO task, that is, the list of choice rules, their order, and possible key responses. After this introduction phase, a short practice block followed, which consisted of three RUPHO tasks with immediate feedback on accuracy after each response. Then, participants were introduced to the letter classification task as the interruption task. In the practice block of this task, participants had to classify each out of six possible letters as a vowel or a consonant and they received immediate feedback after each response. After being introduced to both tasks and the experimental procedure, a knowledge test addressing the RUPHO task (choice rules, their order, and possible answer alternatives) and the experimental procedure was administered. Before starting the test, participants got additional time to memorize again the relevant task information about stimulus and response assignments in the RUPHO task. To pass the knowledge test, participants were required to answer all questions correctly. In case of an error, they received feedback on the specific question and clarifying information about the correct answer. After that participants were asked the critical question again until all answers were correct.

After passing the knowledge test, a baseline block consisting of 32 RUPHO tasks alone was administered. This baseline block was included to ensure that all four groups are comparable with each other concerning the performance in the primary RUPHO task. Then, a final practice block with 10 primary tasks with interruptions took place. When the practice phase was completed, the main experimental session consisting of four experimental blocks started.

Each block consisted of 24 primary RUPHO tasks. Depending on the global frequency group condition, either 25% or 75% of the RUPHO tasks were interrupted, resulting in six or 18 interrupted RUPHO tasks per block, respectively, while the rest remained uninterrupted. In addition, depending on the local frequency group condition, interrupted RUPHO tasks were interrupted either once per multistep task or three times per multistep task. Interruptions occurred with equal probability at four different positions within the RUPHO task (before steps U, P, H, or O)^2^. The order of interrupted and uninterrupted RUPHO tasks was randomized per block and participant. In case of an interruption, the display of the interruption task replaced the display of the RUPHO task immediately and completely. In the interruption task, participants completed 10 trials of the letter classification task in a self-paced manner with an inter-trial interval of 100 ms. After completing the interruption task, the visual display of the RUPHO task was shown immediately again (the same display as before the interruption). Participants were instructed to resume the primary task with the task step that correctly continued the primary task after interruption. After the last step of the RUPHO task was completed, the stimulus of the primary task disappeared, and a new number-stimulus marked the start of a new multistep task after an inter-trial interval of 1500 ms. After completing all experimental blocks, participants filled in a post-experimental questionnaire in which they were asked about the strategies they applied while conducting the task and dealing with interruptions, as well as subjective ratings of the performance in each of the tasks.

### Design

The experimental design for the analysis of the performance in the primary task corresponded to a 4 (Frequency) x 2 (Interruption presence) mixed factorial design. The interruption frequency factor was defined as a between-subjects factor with four levels: the low frequency with 24 interruptions in total (25% of primary tasks interrupted once per task), the 25%-mid frequency with 72 interruptions in total (25% of primary tasks interrupted three times per task), the 75%-mid frequency with 72 interruptions in total (75% of primary tasks interrupted once per task), and the high frequency with 214 interruptions in total (75% of primary tasks interrupted three times). The interruption presence factor was a within-subject factor with two levels: interrupted task and uninterrupted task.

### Dependent variables

To analyze the performance of participants in the primary task we calculated three dependent variables: response times, sequence errors, and non-sequence errors. Performance in the interruption task was analyzed by measuring response times and accuracy rate.

Response time is defined as the time needed to complete a step of the multistep primary task. As interruptions never preceded the first step in the multistep task (R step), only response times to the remaining four steps (steps U, P, H, and O) were averaged and analyzed. In the interrupted primary task, response times were operationalized as the time elapsed between the presentation of the stimulus for the primary task after the interruption again and the first key response. In the uninterrupted primary task, the response times were measured as inter-response intervals for the corresponding steps in the multistep task. Only correct responses were included in this measure.

Sequence errors represent a deviation from the prescribed order of steps in the primary task (i.e., repeating or skipping a step). In the interrupted primary task, it was formally measured in relation to the predefined interruption position, that is, interruptions could occur after steps R, U, P, and H, and steps U, P, H, and O should be answered after these interruptions, respectively. In the uninterrupted primary task, they were formally defined in relation to the preceding step based on the provided response.

Non-sequence errors are defined as errors in the evaluation of a property of a stimulus (e.g., indicating that the number is red instead of blue). Note that in this case, the order of the steps in the sequence may remain intact, that is, not consisting of a sequence error.

In the interruption task, response times are defined as the time needed to classify each letter and it was measured between the letter-stimulus onset and the first response and were used for calculating interruption length.

The correctness rate in the interruption task was defined as a proportion of correctly classified letters as vowels or consonants and was used as a control measure (see *Data processing* section).

### Participants

97 university students (80 female, 14 male, 3 other; *M* = 23.09 years, *SD* = 3.58) took part in the study for monetary compensation or a course credit. A power analysis was calculated using G-power sample size calculator (Faul et al., 2007, 2009) for a within-between interaction effect for ANOVA with two repeated measurements and four groups, *p* = .05, power of 0.95, and partial eta-square of 0.05 based on the previous study on the effects of frequency (Radović et al., 2022). This resulted in a total sample size of 88 participants. Thus, the chosen sample size should suffice after the exclusion of single participants based on their performance or technical issues.

## Results

### Data processing

Data processing was conducted in R-studio (R core team, 2021) using a custom-made script. For the analyses of performance in the primary task, we applied the following exclusion criteria: At the level of individual steps in the primary task, all response times and resumption times faster than 200 ms and longer than 3 *SD* above the group mean per condition (interrupted or uninterrupted) were excluded from the further analyses and these steps were excluded from the calculation of the error rates. In addition, steps with resumption times and post-interruption errors after interruptions that were shorter than 5 s, longer than 10 s, or below the 80% of accuracy were also excluded from further analyses^3^. This resulted in exclusion of 7% of individual data points.

Moreover, due to technical problems, incomplete datasets from three participants were excluded from further analyses. In addition, one participant forgot response keys for one of the steps of the primary task and was excluded from the further analysis.

Finally, in the post-experimental questionnaire about the participants’ strategies used to remember the position in the primary task during the interruption phase, four participants reported using their fingers to mark the keys of the critical steps in the primary task. As participants did not rely on their memory in this case, such strategies were considered as unwanted, and complete datasets of these four participants were excluded. This resulted in a total sample size of 89 participants (23 in the low-frequency group and 22 in each of the remaining three groups).

### Baseline performance in the primary task performance in the interruption frequency groups

To assess potential differences between the experimental groups in terms of baseline performance, we analyzed response times, sequence errors and non-sequence errors from the baseline block containing the uninterrupted RUPHO task only. A one-way ANOVA revealed no differences between the frequency groups in terms of their response times, *F*(3, 85) = .66, *p* = .58, sequence errors, *F*(3, 85) = 0.40, *p* = .76, and non-sequence errors, *F*(3, 85) = 1.27, *p* = .28. These results confirm that the frequency groups were comparable according to their baseline performance.

### Effects of interruption frequency on the performance in the primary task: omnibus analyses

In order to distinguish between the two theoretical approaches and evaluate the proposed hypotheses on the effects of total number of interruptions on the performance in the interrupted and uninterrupted tasks in a direct way, we first conducted a 4 × 2 ANOVA. This analysis corresponds to the proposed hypotheses and is essential because it enables us to directly assess potential performance differences that occur due to a different overall amount of interruptions between the frequency groups (namely, 24 vs. 72 vs. 214 interruptions) with one overall comparison. In addition, such an omnibus analysis allows us also to elaborate in more detail the dynamics of interruption influence on behavior. For example, it allows us to assess at which point of a potential continuum between only a few or many interruptions a potential performance changes may start to emerge (i.e., comparing 24 vs. 72 vs. 214 interruptions).

Based on the MfG theory (Altmann & Trafton, 2002), we expected that with increasing interruption frequency the performance in both interrupted and uninterrupted RUPHO task would improve, as reflected by faster responses and/or less sequence errors. On the other hand, based on the DMC framework (e.g., Braver, 2012), we expect that increasing interruption frequency would have opposite effects on the interrupted and uninterrupted RUPHO task. That is, the performance in interrupted RUPHO task would improve with higher interruption frequency resulting in faster responses and/or less sequence errors, while the performance in the uninterrupted RUPHO task would suffer as interruption frequency increases, reflected in slower responses and/or more errors.

Finally, in order to investigate the potential impact of the level of task interruption, we conducted additional analyses of the effects of the frequency manipulations at the local and global task levels in exploratory fashion. Moreover, we conducted additional analyses in order to control for the potential effect of the amount of practice with interrupted and uninterrupted RUPHO tasks and will report the related findings in the corresponding sections below.

#### Response times

**Fig. 2:**
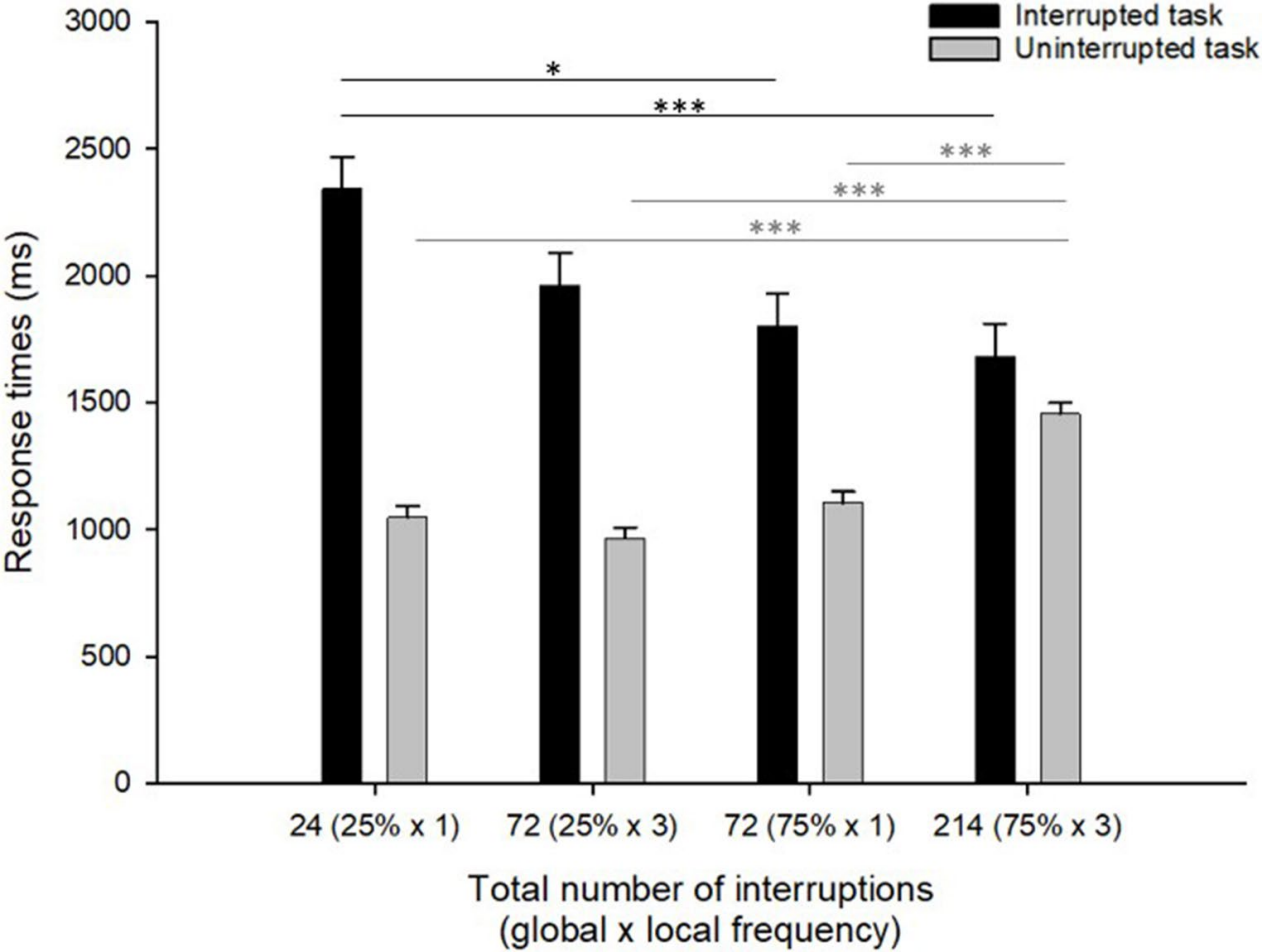
Reaction times (in ms) as a function of interruption presence (black bars as interrupted task; gray bars as uninterrupted task) and the total number of interruptions (x-axis; variation in the global and local interruption frequency is presented in brackets): 24 interruptions at all (25% of RUPHO tasks, which were interrupted once), 72 interruptions at all (25% of RUPHO tasks, which were interrupted three times), 72 interruptions at all (75% of the RUPHO tasks, which were interrupted once), and 214 interruptions at all (75% of the RUPHO tasks, which were interrupted three times). Error bars represent the standard error of the mean. Note. Significant post-hoc tests between the frequency groups are represented in the horizontal lines: in black for the interrupted tasks and in grey for the uninterrupted tasks. *p<.05. ***p<.001

Mean response times for the different frequency groups on interrupted and uninterrupted RUPHO tasks are presented in Fig. 2. A visual inspection of the data indicates that responses in the interrupted RUPHO tasks (Fig. 2, black bars) seem generally slower than responses in the uninterrupted RUPHO tasks (Fig. 2, gray bars) for all frequency groups. Concerning our main research question, it seems that as the interruption frequency increases (Fig. 2, x-axis from left to right) responses in the interrupted RUPHO tasks are becoming faster (Fig. 2, black bars), while the responses in the uninterrupted RUPHO tasks are becoming slower (Fig. 2, gray bars).

This impression was confirmed by the results of the corresponding analyses of variance (ANOVA). Thus, an analysis of response times in the RUPHO task with a 4 (Frequency) x 2 (Interruption presence) mixed ANOVA revealed a main effect of the interruption presence, *F*(1, 85) = 191.08, *p* < .001, η² = .69. Specifically, response times in the interrupted RUPHO tasks were significantly higher than response times in the uninterrupted RUPHO tasks, which is in line with the previous studies (e.g., Altmann et al., 2014; Radović & Manzey, 2019). At the same time, the effect of the frequency condition was not significant, *F*(3, 85) = 2.32, *p* = .08, η² = .08. However, most importantly, we found a significant interaction between interruption frequency and interruption presence on response times, *F*(3, 85) = 15.49, *p* < .001, η² = .35, which confirms the observation of the opposite effect pattern of interruption frequency on the response times in the interrupted RUPHO and the uninterrupted RUPHO tasks.

Specifically, in the interrupted RUPHO tasks, post-hoc tests with Bonferroni correction revealed significantly slower responses in the low-frequency group with 24 interruptions (*M* = 2344 ms, *SE* = 125) compared to the 75% group with 72 interruptions (*M* = 1801 ms, *SE* = 128, *p* = .019), and the high-frequency group with 214 interruptions (*M* = 1682 ms, *SE* = 128, *p*<.001). No other differences between the groups were significant (all *ps* > .21). In comparison, in the uninterrupted RUPHO tasks, post-hoc tests with Bonferroni correction revealed slower responses in the high-frequency group with 214 interruptions (*M* = 1456 ms, *SE* = 43) than in the other three groups with lower interruption frequencies: the low-frequency group with 24 interruptions (*M* = 1048 ms, *SE* = 42), the 25% group with 72 interruptions (*M* = 966 ms, *SE* = 43), and the 75% group with 72 interruptions (*M* = 1105 ms, *SE* = 43), all *ps* < .001. No other differences between the groups were significant (all *ps* > .14). Thus, in contrast to the result pattern observed in the interrupted RUPHO task, in the uninterrupted RUPHO task we found slower performance when the interruption frequency is high.

#### Sequence errors

**Fig. 3:**
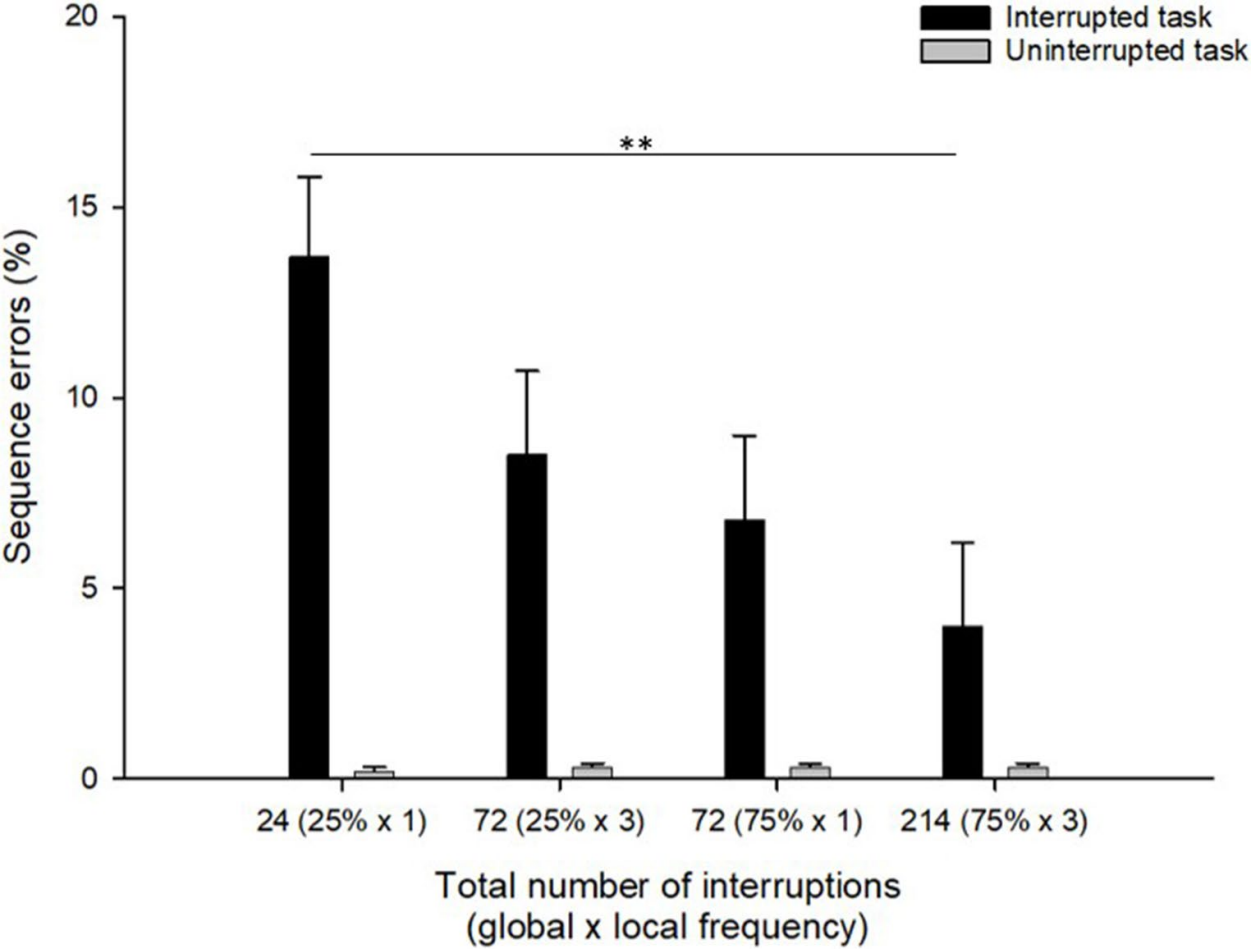
Rates of sequence errors (in %) as a function of interruption presence (black bars as interrupted task; gray bars as uninterrupted task) and the total number of interruptions (x-axis; variation in the global and local interruption frequency is presented in brackets): 24 interruptions (25% of tasks interrupted once), 72 interruptions (25% of tasks interrupted three times), 72 interruptions (75% of tasks interrupted once), and 214 interruptions (75% of tasks interrupted three times). Error bars represent the standard error of the mean. Note. Significant post-hoc tests between the frequency groups are represented in the horizontal line in black for the interrupted tasks. **p<.01

Mean rates of sequence errors for the different frequency groups on interrupted and uninterrupted RUPHO tasks are presented in Fig. 3. A visual inspection of the data shows that the rates of sequence errors in the interrupted RUPHO tasks (Fig. 3, black bars) are higher than in the uninterrupted RUPHO tasks (Fig. 3, gray bars) for all frequency groups. Regarding our main research question, it seems that as interruption frequency increases (Fig. 3, x-axis from left to right) participants make less sequence errors in the interrupted RUPHO tasks (Fig. 3, black bars), while the uninterrupted RUPHO tasks rates of sequence errors remain very low for all groups (Fig. 3, gray bars).

This impression is confirmed by the results of a 4 (Frequency) x 2 (Interruption presence) mixed ANOVA. This analysis showed a main effect of the interruption presence on sequence errors, *F*(1, 85) = 56.35, *p* < .001, η² = .40; participants made significantly more sequence errors in the interrupted than in the uninterrupted RUPHO tasks replicating again the detrimental effect of interruption presence on sequence errors from previous studies (e.g., Altmann et al., 2014; Radović & Manzey, 2019). In addition, we also found a significant effect of interruption frequency, *F*(3, 85) = 3.49, *p* = .02, η² = .11, and, critically, an interaction effect between interruption presence and interruption frequency, *F*(3, 85) = 3.83, *p* = .01, η² = .12, on the rate of sequence errors. The latter reflects the observation of a different result pattern for the rates of sequence errors in the interrupted and uninterrupted RUPHO tasks. Specifically, for the interrupted RUPHO task, post-hoc tests with Bonferroni correction revealed more errors in the low-frequency group with 24 interruptions (*M* = 13.7%, *SE* = 2.1) than in the high-frequency group with 214 interruptions (*M* = 4%, *SE* = 2.2, *p* = .01), while no other differences between the groups were significant (all *ps* > .15). With respect to the performance in uninterrupted RUPHO tasks, the rates of sequence errors were generally very low (below 1% in each group) and no significant differences occurred between different interruption frequency groups (all *ps* > .85).

#### Non-sequence errors

Rates of non-sequence errors were analyzed using a 4 (Frequency) x 2 (Interruption presence) mixed ANOVA. This analysis did not reveal any significant main effect and no significant interaction between the factors on the occurrence of non-sequence errors, all *ps* > .41. As no effects were obtained in the rates of non-sequence errors, this dependent variable will be skipped from additional analyses reported in the sections below. As response times and sequence errors were affected by the presence of interruptions and the rate of non-sequence errors were not, the current results stand in line with those of previous studies that showed specific effects of interruptions on participants’ performance in a serial multistep primary task (e.g., Altmann et al., 2014; Radović & Manzey, 2022). This indicates the validity of the current RUPHO task for investigating the potential effects of interruptions on task and resumption performance.

To sum up, the results of the omnibus analyses suggest that increasing interruption frequency has opposite effects on the performance in the interrupted and uninterrupted RUPHO tasks. Specifically, with higher interruption frequency performance improved in the interrupted RUPHO tasks, as reflected in faster responses and lower rates of sequence errors, while performance became slower in the uninterrupted RUPHO tasks under conditions of a higher interruption frequency.

### Additional analyses: Effects of interruption frequency when controlling for different amounts of practice with interruptions

Participants in the different frequency groups had different amount of practice for performing the interrupted RUPHO task and the uninterrupted RUPHO task. Specifically, in the low frequency group participants encountered 24 interruptions in total, in the 75%-group and the 25%-group participants encountered 72 interruptions in total and in the high frequency group they dealt with 214 interruptions in total. To investigate whether the obtained effects of interruption frequency can be explained by such a practice effect, we conducted additional analyses separately for interrupted and uninterrupted RUPHO tasks. To control for the different amount of practice with interruptions across the different groups, we analyzed the performance of participants in a subset of tasks, which covered the first 23 interruptions in the RUPHO task and represented the smallest number of interruptions identical for all frequency groups. Please note that this number was set to 23 instead of 24 interruptions because several participants in the low frequency group with 24 interruptions occasionally committed a sequence error in the primary task and this reduced the total number of interruptions from 24 to 23 for this group. Per analogy, for controlling for the potential effect of practice on the performance of the different frequency groups with the uninterrupted RUPHO tasks, we compared the performance of the different frequency groups for the first 24 uninterrupted RUPHO tasks. Namely, if we find again significant differences between the frequency conditions (while controlling for differences in amount of practice), then this would suggest that anticipation of interruption frequency and preparation to deal with frequent interference are driving the above-mentioned differences instead of the amount of practice with both uninterrupted and interrupted the primary tasks.

#### Interrupted performance

Response times. We analyzed mean response times after the first 23 interruptions in the RUPHO task using a one-way ANOVA with frequency group as a factor. When controlling for the amount of practice with interruptions, the results did not reveal any significant differences between the four frequency groups in terms of response times in the interrupted tasks, *F*(3, 85) = 1.36, *p* = .26. In other words, the above-mentioned differences between the frequency groups in response times in the interrupted tasks disappeared after controlling for the different amount of practice with interruptions.

Sequence errors. A one-way ANOVA with frequency condition as a factor revealed a significant effect of interruption frequency on the sequence errors, *F*(3, 85) = 2.88, *p* = .04, η² = .09. As reported in the previous section, post-hoc tests with Bonferroni correction revealed that this effect was driven by lower rates of sequence errors in the high-frequency group compared to the low-frequency group (*M* = 4.9%, *SE* = 1.1 vs. *M* = 14.2%, *SE* = 3, *p* = .03). Thus, the occurrence of a lower sequence error rate at resumption in the high-frequency group compared to the low-frequency group remains a stable finding even if we control for the different amount of practice with interruptions between groups in the RUPHO task.

#### Uninterrupted performance

Response times. An ANOVA with frequency group as factor confirmed the above-reported differences between the frequency groups in terms of response times for the first 24 uninterrupted RUPHO tasks, *F*(3, 85) = 12.68, *p* < .001, η² = .31. Again, post-hoc tests with Bonferroni correction revealed that participants in the high-frequency group with 214 interruptions needed more time to complete a step in the primary task (*M* = 1468 ms, *SE* = 47) compared to the other three groups: the low-frequency group with 24 interruptions (*M* = 1271 ms, *SE* = 35, *p* = .003), the 25% group with 72 interruptions (*M* = 1155 ms, *SE* = 20, *p* < .001) and the 75% group with 72 interruptions (*M* = 1188 ms, *SE* = 47, *p* < .001). No other differences between the groups were significant (all *p*s > .11). This finding suggests that the slower responses in the uninterrupted tasks found in the high-frequency group cannot be attributed to less practice of this group with the uninterrupted RUPHO task.

Sequence errors. As rates of sequence errors in the interrupted RUPHO tasks remained low (< 1%) in all frequency groups, they were not analyzed further.

To sum up, when controlling for a potential effect of the different amount of practice with the occurrence of interrupted and uninterrupted RUPHO tasks across the different frequency groups, the results confirmed the above-reported pattern of findings at least partly. First, with respect to the interrupted RUPHO task, the results show that the above-reported positive effect of high interruption frequency on response times in RUPHO task was driven by the increased amount of practice with the occurrence of interruptions during task processing. Nevertheless, the fact that the observed positive effect of high interruption frequency on the rate of sequence errors remained even when we controlled for the different amount of practice between groups suggests a strategic shift towards better dealing with interruptions during task processing. Second, with respect to the uninterrupted RUPHO task, the results indicated that high interruption frequency leads to slower responses not because of less practice with this type of task, as the main pattern of results was preserved after controlling for different amount of practice. Probably, the strategic shift towards dealing with interruptions comes at the cost of slower performance in the uninterrupted task for the high frequency group compared to the other groups. This assumption will be further elaborated in the General discussion.

### Additional analyses: assessing potential effects of global interruption frequency and local interruption frequency

In the present study, interruption frequency varied simultaneously at the global level (i.e., number of RUPHO tasks interrupted at all) and the local level (i.e., number of interruptions within one multistep RUPHO task). Therefore, we aimed to assess in more detail whether or not the frequency of interruptions affects task processing in different ways, if manipulated on the global or on the local level of task processing. Thus, we conducted two additional 2 (Global frequency: low vs. high) x 2 (Local frequency: low vs. high) between-subjects ANOVAs separately for the performance in the interrupted and in the uninterrupted the RUPHO task.

#### Interrupted RUPHO task

Response times. As can be seen in Fig. 2, participants in the global high frequency condition (75% of RUPHO tasks interrupted; two black bars on the right side) responded faster than the participants in the global low frequency conditions (25% of RUPHO tasks interrupted; two black bars on the left side). At the same time, participants in the local high frequency condition (three interruptions per RUPHO multistep task) responded faster than participants in the local low frequency condition (one interruption per RUPHO multistep task). This is reflected by the results of the 2 × 2 ANOVA, which revealed a main effect of global interruption frequency on response times, *F*(1, 85) = 10.48, *p* = .002, η² = .11, with faster responses in the two 75%-conditions (*M* = 1742 ms, *SE* = 90) compared to the two 25%-frequency conditions (*M* = 2153 ms, *SE* = 89). The ANOVA revealed also a main effect of local interruption frequency on response times, *F*(1, 85) = 3.89, *p* = .05, η² = .04, with faster performance in the RUPHO tasks if interrupted three times (*M* = 1822 ms, *SE* = 90) compared to interrupted once (*M* = 2073 ms, *SE* = 89), while the interaction between the two factors was not significant, *F*(1, 85) = 1.07, *p* = .30. To sum up, this analysis showed that the faster resumption of the interrupted RUPHO task was driven by both high interruption frequency at the global level and the local level in additive manner.

Sequence errors. As can be seen in Fig. 3, participants in the global high frequency conditions (75% of RUPHO tasks interrupted; two black bars on right side) made less sequence errors than the participants in the global low frequency conditions (25% of RUPHO tasks interrupted; two black bars on left side). The corresponding ANOVA revealed a main effect of global interruption frequency on sequence errors, *F*(1, 85) = 7.03, *p* = .01, η² = .08, with less errors in the global high frequency condition (*M* = 5.4%, *SE* = 1.5) compared to the global low frequency condition (*M* = 11.1%, *SE* = 1.5). The effect of local interruption frequency on sequence errors approached significance, *F*(1, 85) = 3.49, *p* = .065, with a tendentially smaller rate of sequence errors in the local high frequency conditions with three interruptions per RUPHO task (*M* = 6.3%, *SE* = 1.5) compared to the local low frequency condition with one interruption per task (*M* = 10.3%, *SE* = 1.5). The interaction between the global and local frequency factors was not significant, *F*(1, 85) = .32, *p* = .58.

#### Uninterrupted performance

Response times. A closer visual inspection of Fig. 2 indicates increasing response times in the uninterrupted RUPHO task with increasing frequency of interruptions. However, this increase seems especially expressed if both, global interruption frequency and local interruption frequency are high (75% of tasks interrupted three times, gray bar on the right side). The is reflected by the 2 × 2 ANOVA, which revealed a main effect of global interruption frequency, *F*(1, 85) = 41.58, *p* < .001, η² = .33, a main effect of local interruption frequency, *F*(1, 85) = 9.99, *p* = .002, η² = .11, and, critically, an interaction between the global and local frequency factors, *F*(1, 85) = 26.13, *p* < .001, η² = .24. Post-hoc tests with Bonferroni correction revealed significantly higher response times in the uninterrupted RUPHO task when both global and local interruption frequencies were high (75% of RUPHO tasks interrupted three times; *M* = 1456 ms, *SE* = 43) compared to all other frequency conditions: low global x low local frequency (25% of RUPHO tasks interrupted once; *M* = 1048 ms, *SE* = 54, *p* < .001), low global x high local frequency condition (25% of RUPHO tasks interrupted three times; *M* = 966 ms, *SE* = 43, *p* < .001), and the high global x low local frequency condition (75% of RUPHO tasks interrupted once; *M* = 1105 ms, *SE* = 43, *p* < .001). No other differences between the groups were significant (all *p* > .17). To sum up, this analysis showed that the slowing down in the uninterrupted RUPHO task is especially strong when both, global interruption frequency and local interruption frequency, were high.

Sequence errors. Due to the low rate of errors, we did not analyze them in the uninterrupted tasks.

## Discussion

The goal of the present study was to investigate the effects of interruption frequency on interrupted and uninterrupted performance in a serial multistep primary task. In addition, the subordinate goal of the current research was to disentangle the effects of different types of manipulation of interruption frequency. Therefore, we systematically manipulated interruption frequency at the global level, as a percentage of interrupted tasks at all (25% vs. 75%), and at the local level, as the number of interruptions per task (one or three interruptions).

Most importantly, the current results suggest that increasing interruption frequency has opposite effects on the performance in the interrupted and uninterrupted RUPHO task. Specifically, with higher interruption frequency the performance improved in the interrupted RUPHO tasks, as reflected in faster responses and lower rates of sequence errors with increasing interruption frequency. In contrast to the improved performance in the interrupted tasks, when it comes to performance in uninterrupted RUPHO tasks, under very high interruption frequency responses were becoming slower. Moreover, this effect of slowing down emerges especially in the situation when both global and local interruption frequency were high, which suggests dose-dependent effects of interruption frequency.

Generally, the findings of positive effects of high interruption frequency on the resumption performance are in line with the results of some of the previous research that also manipulated the frequency on the global level and found faster (e.g., Monk, 2004; Powers & Scerbo, 2022; Radović et al., 2022) and more accurate resumption performance, at least descriptively (e.g., Monk, 2004). However, discussing the result of negative effect of high interruption frequency in the uninterrupted tasks in the context of the previous research is challenging, because earlier studies have addressed the potential effects of interruptions on the performance in the uninterrupted primary task rather rarely. In fact, two earlier studies have at least suggested the opposite pattern, that is, that higher interruption frequency might lead to an improvement in uninterrupted performance as well (Radović et al., 2022; Zijlstra et al., 1999). In these previous studies, however, total number of interruptions was rather low (e.g., up to 72 interruptions in total when 75% of tasks were interrupted once; Radović et al., 2022) compared to the current study (214 interruptions). Thus, it might be the case that the detrimental effects of interruption frequency on the uninterrupted performance were not able to emerge under the relatively narrow frequency range applied in the previous studies, which is in line with the assumption of dose-dependent effects of interruption frequency. In addition, it is tempting to assume that interrupting a task above a certain threshold (i.e., above a certain number of interruptions) drives the detrimental effect of high interruption frequency on the performance in the uninterrupted tasks. Besides the differences in total amount of interruptions, these previous studies (e.g., Monk, 2004; Radović et al., 2022; Zijlstra et al., 1999) differed in terms of the type of the primary tasks which they administered. In the current study, the primary task was a demanding, memory-based multistep task, while the previous research applied a visual search task (Radović et al., 2022) consisting of only one goal (i.e., to find a target letter among distractors). Thus, it seems plausible that the overall cognitive demands of the tasks might be decisive for the occurrence of the discrepant results across studies. In more detail, under relatively low cognitive demands the high interruption frequency might lead to faster performance in the task by increasing arousal (Baethge et al., 2015). However, as cognitive demands increase above a certain threshold, through task complexity and high interruption frequency, the cognitive system may be forced to operate at or beyond its cognitive limits. Therefore, the system is enforced to optimize the processing via a strategic shift towards dealing with frequent interference at a cost of worse performance in the uninterrupted tasks where no such interference is present (for further elaboration see the paragraph below). We are aware that an assumption of a threshold resulting from the interaction of task complexity and interruption frequency is rather speculative and needs further investigation. Nonetheless, it can empirically be tested because it predicts that with even higher complexity of the primary task and/or interruption task, the strategic processing shift could emerge even under lower interruption frequency than in the present study and this should be reflected in better resumption performance and impaired uninterrupted performance.

Taken together, the pattern of results revealed that increasing interruption frequency led to faster and more accurate resumption after an interruption, but also to slower responses in the uninterrupted task. This pattern of results is generally in line with the predictions of the DMC framework, which postulates two modes of cognitive control, proactive mode and reactive mode (Braver, 2012). To which degree each of these modes will be involved in task processing depends on the frequency of interfering events. On one hand, if interference from another task is relatively rare, the reactive mode of cognitive control would be employed when dealing with this unexpected interference. Resolving the interference in such a post-hoc manner, that is, after its onset, would be required in situations with low interruption frequency compared to situations where interruptions occur more often. On the other hand, if interference from another task is frequent, a proactive mode of control would be recruited in order to prepare for the expected interference and adjust the cognitive system accordingly. This preparation might involve different cognitive processes, such as more efficient encoding and/or retrieval of the last completed goal of the primary task before the interruption, reconstruction of the task space of the primary task or retrieval of the currently relevant task-goal. Thus, such a strategic shift can be beneficial when dealing with interference caused by interruptions which can explain the faster and more accurate resumption performance under situations with high interruption frequency. At the same time, such preparation for dealing with interruptions would not be useful in the rare situations in which the interruption is not present. Thus, when the uninterrupted task is processed in the context of high interference, the performance in this task will suffer and result in slower responses in the task.

Besides the strategic shift in cognitive control, the positive effects of high interruption frequency on the performance in the interrupted primary task seem to emerge also due to more practice with interruptions. Importantly, when we controlled for the amount of practice of dealing with interruptions, the differences in resumption times between the frequency conditions diminished. This suggests that the observed speed-up in the resumption performance was driven to some extent by the different amount of practice that participants in different frequency groups were exposed to. However, differences in sequence errors between the frequency conditions remained constant even when the amount of practice of dealing with interruptions was equalized across groups. This implies that anticipation of the high probability of interruptions led to proactive preparation to deal with this interference and not the amount of practice with interrupted tasks. Overall, this suggests that the positive effects of higher interruption frequency on the resumption performance are realized by different mechanisms. In more detail, both previous practice with interruptions and a strategic shift in the mode of cognitive control toward dealing with interruptions (the DMC framework, Braver, 2012) seem to play a role for different aspects of resumption performance by improving resumption times and reducing error rates, respectively.

In addition, to the best of our knowledge, the effects of local and global interruption frequency on performance were investigated systematically for the first time by the current study. Namely, in the previous studies manipulation of local interruption frequency was often confounded with global interruption frequency, as interrupting a single task multiple times (high local frequency) resulted also in more interruptions overall (high global frequency; e.g., Speier et al., 1999). In addition, it seems plausible that manipulating local and global interruption frequency independently of each other could affect processing of goals of the multistep primary task in a different way. For example, according to Altmann and colleagues (2017) representation of a multistep task is stored in semantic memory and performing any step from this task automatically primes all following steps due to spreading activation gradually declining from one step to the next. As spreading of activation from the last completed subgoal of the task to the next would be disrupted when the task is interrupted three times, larger resumption costs would arise compared to a single interruption, despite the total number of interruptions is constant (e.g., 25%- and 75%-groups each with 72 interruptions). However, our results did not provide evidence for this assumption. In fact, local interruption frequency and global interruption frequency seem to affect resumption performance in the same direction and independently of each other, as each led to faster resumption under high frequency condition than in the low frequency condition. Interestingly, more accurate resumption seems to be predominantly related to high interruption frequency at the global level, while a tendency in the same direction was shown under high local interruption frequency. The fact that we found similar and independent effects of the variation of local interruption frequency and global interruption frequency on resumption performance suggests that interrupting a multistep task (i.e., RUPHO task) more often at a local level (i.e., within one task) or at the global level (once per task over more tasks) leads to similar dynamics of goal re-activation. This further implies that for the spreading activation mechanism to enhance processing of the next step in the multistep task, it is sufficient that a previous subgoal of the task is sufficiently activated or re-activated (in case of a resumption) and that the effects of consecutive interruptions do not accumulate over time, additionally impairing this process. As discussed above, increasing both local and global frequency at the same time leads to a condition with very high interruption frequency, which has a detrimental effect on the uninterrupted performance. Generally, the current findings suggest that the total number of interruptions is a decisive factor affecting performance in a multistep task. Nevertheless, future studies might further disentangle whether interruptions at different task levels might show differential effects on the performance in the interrupted and uninterrupted tasks. While in the present study we aimed to ensure comparability in the interruption task performance between different frequency groups, another potential avenue for future research would be to examine differences in interruption-task performance across different interruption-frequency conditions. Such an approach would allow to directly assess whether higher interruption frequency also leads to faster and more accurate interruption-task performance, as predicted by the MfG framework.

Moreover, for the purpose of this study, we administered a novel multistep task as a primary task (i.e., the RUPHO task), which was constructed in line with other serial multistep primary tasks used in previous research on task interruptions (e.g., Altmann et al., 2014; Hirsch et al., 2023; Kopacz et al., 2019; Radović & Manzey, 2019, 2022). Using the RUPHO task, we replicated the standard finding that interruptions lead to costs specifically in terms of response times and sequence errors when resuming the primary task after an interruption (e.g., Altmann et al., 2014; Hodgetts & Jones, 2006; Kopacz et al., 2019; Monk et al., 2008; Radović & Manzey, 2019). Namely, completing a task step in the RUPHO task directly after an interruption took more time and it was more likely to skip or repeat a step in the multistep task compared to the primary tasks where no interruption was present, reflecting resumption costs. At the same time, the rates of non-sequence errors were not affected by interruptions. This finding stands in line with the results of the previous research using various multistep tasks and shows the specific effect of interruptions on goal activation in memory (e.g., Altmann et al., 2014; Kopacz et al., 2019; Radović & Manzey, 2019). Namely, these resumption costs can be explained by the MfG theory (Altmann & Trafton, 2002). According to this theory, task goals must be activated in working memory to perform a multistep task, thus active strengthening is required to achieve a sufficient level of activation for successful retrieval of these goals (i.e., above an interference level). Interruptions during a multistep task lead to a decay in the activation of the goals of this interrupted task. Therefore, to resume the multistep task at the correct point after an interruption, one must re-elevate the activation level of the goal associated with the correct task step at the point of resumption. This process of re-activation is time-consuming, leading to longer resumption times. This process is also error-prone, as it increases the probability of retrieving the wrong goal (i.e., step) of the multistep task, resulting in a higher probability of committing a sequence error. The current study expanded these previous findings showing that the size of these resumption costs is modulated by variations in interruption frequency.

In general, the findings indicate that the efficiency of resuming a task after an interruption represents a trainable cognitive skill rather than a necessary performance limitation. Repeated exposure to interruptions as well as preparation mechanisms appear to foster adaptive mechanisms that enhance individuals’ ability to resume primary tasks efficiently, suggesting that the negative effects of interruptions can be mitigated through practice and preparation, at least partly (for a review see Guo et al., 2021). This seems to be particularly relevant for occupational domains in which interruptions are both frequent and unavoidable, including healthcare (e.g., Drews et al. 2019; Westbrook et al. 2010a, b), air traffic control (Latorella, 1996; Wilson et al., 2018), and office work (e.g., Zijlstra et al., 1999). Instead of prioritizing the elimination of interruptions (e.g., Westbrook et al., 2017), training interventions focusing on practice as well as preparation to deal with interference should be designed to simulate high-interruption contexts, enabling practitioners to acquire and refine effective resumption strategies under ecologically valid conditions (for a review see Raban & Westbrook, 2014).

## Conclusions

The present study addressed the issue of interruption frequency in a serial multistep task. Namely, the positive effects of interruption frequency on the resumption performance were also found in a multistep task with a fixed order of steps that have to be learned and then performed from memory. The variation of interruption frequency had a specific effect on goal processing during the resumption of the task, which was previously shown for the other properties of interruption tasks, such as interruption length (e.g., Altmann et al., 2014, 2017; Radović & Manzey, 2019) or complexity of an interruption task (e.g., Radović & Manzey, 2022). Besides influencing the resumption performance, the variation in interruption frequency played a role indirectly when the task is performed without any interruptions.

We observed opposite effects of interruption frequency on performance in interrupted and uninterrupted tasks, that is, resumption performance was improved under high interruption frequency and simultaneously deteriorates when the task is not interrupted. Moreover, the improvement in resumption performance under high interruption frequency is supported by different cognitive mechanisms. Partly, these effects can be explained by more practice with interruptions. Also, the positive effect of high interruption frequency seems to be mediated by a shift in processing mode and a more strategic preparation to deal with interruptions. While such preparation is beneficial for one aspect of performance, that is, resumption accuracy, it could also account for slower progress in the uninterrupted task. This shift toward a more proactive processing mode of cognitive control seems to be caused by the variation of interruption frequency predominantly on the global level.

The findings of our study suggest an account for the inconsistent results of the previous studies, at least partly. For example, if a resumption cost, often calculated as a difference in the performance between the interrupted and uninterrupted tasks, or some other derivative measure of performance is analyzed, the results might even reveal no negative effects of interruptions on the performance in a primary task (especially when the frequency is very high). However, this potential lack of effect might not be due to a general improvement of the resumption performance only, but due to the negative effect in the uninterrupted tasks. Thus, the present study is in favor of evaluating the effects of interruptions and their properties on the interrupted and uninterrupted primary tasks separately in future research.

## Data Availability

Data that support the findings of this study have been deposited in the OSF and can be accessed at DOI 10.17605/OSF.IO/JMHKQ.

## Footnotes

1 Numbers 1, 2, 8, and 9 are peripheral, and numbers 3, 4, 6, and 7 are central to number 5.

2 This means that in the multistep task interrupted once, the interruption task occurred either before step U, or step P, or step H, or step O. If a multistep task was interrupted three times, interruptions occurred either before steps U, P and H, or before steps U, P and O, or before steps P, H, and O, or before steps U, H and O.

3 This cleaning criterion resulted in comparable interruption length in the interruption task between the groups: 7.7 s (low frequency group with 24 interruptions) vs. 7.2 Sect.  (25%-group with 72 interruptions) vs. 7.2 Sect.  (75%-group with 72 interruptions) vs. 7.1 s (high frequency group with 214 interruptions). Also, mean accuracy was comparable between the frequency groups (97.8% vs. 97.6% vs. 97.6% vs. 96.2%, respectively).

## References

[CR1] Altmann, E. M., & Hambrick, D. Z. (2017). Practice increases procedural errors after task interruption. *Journal of Experimental Psychology: General*, *146*(5), 615–620. 10.1037/xge0000274 28301178 10.1037/xge0000274

[CR4] Altmann, E. M., & Trafton, J. G. (2002). Memory for goals: An activation-based model. *Cognitive Science*, *26*(1), 39–83. 10.1207/s15516709cog2601_2

[CR3] Altmann, E. M., Trafton, J. G., & Hambrick, D. Z. (2014). Momentary interruptions can derail the train of thought. *Journal of Experimental Psychology: General*, *143*(1), 215–226. 10.1037/a0030986 23294345 10.1037/a0030986

[CR2] Altmann, E. M., Trafton, J. G., & Hambrick, D. Z. (2017). Effects of interruption length on procedural errors. *Journal of Experimental Psychology: Applied*, *23*(2), 216–229. 10.1037/xap0000117 28150961 10.1037/xap0000117

[CR5] Baethge, A., Rigotti, T., & Roe, R. A. (2015). Just more of the same, or different? An integrative theoretical framework for the study of cumulative interruptions at work. *European Journal of Work and Organizational Psychology*, *24*(2), 308–323. 10.1080/1359432X.2014.897943

[CR6] Basoglu, K. A., Fuller, M. A., & Sweeney, J. T. (2009). Investigating the effects of computer mediated interruptions: An analysis of task characteristics and interruption frequency on financial performance. *International Journal of Accounting Information Systems*, *10*(4), 177–189. 10.1016/j.accinf.2009.10.003

[CR7] Bonnin, C. A., Gaonac’h, D., & Bouquet, C. A. (2011). Adjustments of task-set control processes: Effect of task switch frequency on task-mixing and task-switching costs. *Journal of Cognitive Psychology*, *23*(8), 985–997. 10.1080/20445911.2011.594435

[CR8] Braver, T. S. (2012). The variable nature of cognitive control: a dual mechanisms framework. *Trends in Cognitive Sciences*, *16*(2), 106–113. 10.1016/j.tics.2011.12.010 22245618 10.1016/j.tics.2011.12.010 PMC3289517

[CR9] Braver, T. S., & Barch, D. M. (2002). A theory of cognitive control, aging cognition, and neuromodulation. *Neuroscience & Biobehavioral Reviews*, *26*(7), 809–817. 10.1016/S0149-7634(02)00067-2 12470692 10.1016/s0149-7634(02)00067-2

[CR10] Cades, D. M., Boehm-Davis, D. A., Trafton, J. G., & Monk, C. A. (2011). Mitigating disruptive effects of interruptions through training: What needs to be practiced? *Journal of Experimental Psychology: Applied*, *17*(2), 97–109. 10.1037/a0023497 21517204 10.1037/a0023497

[CR11] Couffe, C. L., & Michael, G. A. (2017). Failures due to interruptions or distractions: A review and a new framework. *American Journal of Psychology*, *130*(2), 163–181. 10.5406/amerjpsyc.130.2.0163 29461714 10.5406/amerjpsyc.130.2.0163

[CR12] Dodhia, R. M., & Dismukes, R. K. (2009). Interruptions create prospective memory tasks. *Applied Cognitive Psychology*, *23*(1), 73–89. 10.1002/acp.1441

[CR13] Dreisbach, G., & Haider, H. (2006). Preparatory adjustment of cognitive control in the task switching paradigm. *Psychonomic Bulletin & Review*, *13*, 334–338. 10.3758/BF03193853 16893004 10.3758/bf03193853

[CR14] Drews, F. A., Markewitz, B. A., Stoddard, G. J., & Samore, M. H. (2019). Interruptions and delivery of care in the intensive care unit. *Human Factors*, *61*(4), 564–576. 10.1177/0018720819838090 30945959 10.1177/0018720819838090

[CR15] Einstein, G. O., McDaniel, M. A., Williford, C. L., Pagan, J. L., & Dismukes, R. K. (2003). Forgetting of intentions in demanding situations is rapid. *Journal of Experimental Psychology: Applied*, *9*(3), 147–162. 10.1037/1076-898X.9.3.147 14570509 10.1037/1076-898X.9.3.147

[CR17] Faul, F., Erdfelder, E., Lang, A. G., & Buchner, A. (2007). G* Power 3: A flexible statistical power analysis program for the social, behavioral, and biomedical sciences. *Behavior Research Methods*, *39*(2), 175–191. 10.3758/BF03193146 17695343 10.3758/bf03193146

[CR16] Faul, F., Erdfelder, E., Buchner, A., & Lang, A. G. (2009). Statistical power analyses using G* Power 3.1: Tests for correlation and regression analyses. *Behavior Research Methods*, *41*(4), 1149–1160. 10.3758/BRM.41.4.1149 19897823 10.3758/BRM.41.4.1149

[CR18] Grundgeiger, T., & Sanderson, P. (2009). Interruptions in healthcare: theoretical views. *International Journal of Medical Informatics*, *78*(5), 293–307. 10.1016/j.ijmedinf.2008.10.001 19081295 10.1016/j.ijmedinf.2008.10.001

[CR19] Grundgeiger, T., Sanderson, P. M., Oriehuela, C. B., Thompson, A., MacDougall, H. G., Nunnink, L. (2010a). Distractions and interruptions in the intensive care unit: A field observation and a simulator experiment. *Proceedings of Human Factors and Ergonomics Society Annual Meeting* (pp. 835–839). CA, Los Angeles. 10.1177/154193121005401206

[CR20] Grundgeiger, T., Sanderson, P. M., MacDougall, H. G., & Venkatesh, B. (2010b). Interruption management in the intensive care unit: Predicting resumption times and assessing distributed support. *Journal of Experimental Psychology: Applied*, *16*(4), 317. 10.1037/a0021912 21198250 10.1037/a0021912

[CR21] Guo, J., Chen, T., Xie, Z., & Or, C. K. (2021). Effects of interventions to reduce the negative consequences of interruptions on task performance: A systematic review, meta-analysis, and narrative synthesis of laboratory studies. *Applied Ergonomics*, *97*, 103506. 10.1016/j.apergo.2021.103506 34273814 10.1016/j.apergo.2021.103506

[CR22] Hirsch, P., Moretti, L., Askin, S., & Koch, I. (2023). Examining the cognitive processes underlying resumption costs in task-interruption contexts: Decay or inhibition of suspended task goals? *Memory & Cognition*, *52*(2), 271–284. 10.3758/s13421-023-01458-8 37674056 10.3758/s13421-023-01458-8 PMC10896823

[CR23] Hirsch, P., Koch, I., & Grundgeiger, T. (2024). Task interruptions. In A. Kiesel et al. (Eds.), *Handbook of Human Multitasking* (pp. 145–188). Cham, Switzerland: Springer. 10.1007/978-3-031-04760-2_4.

[CR24] Hodgetts, H. M., & Jones, D. M. (2006). Interruption of the Tower of London task: Support for a goal-activation approach. *Journal of Experimental Psychology: General*, *135*(1), 103–115. 10.1037/0096-3445.135.1.103 16478319 10.1037/0096-3445.135.1.103

[CR25] Kopacz, A., Biele, C., & Zdrodowska, A. (2019). Development and validation of a shortened language-specific version of the UNRAVEL placekeeping ability performance measuring tool. *Advances in Cognitive Psychology*, *15*(4), 256–265. 10.5709/acp-0273-3 32494312 10.5709/acp-0273-3 PMC7251942

[CR26] Latorella, K. A. (1996). Investigating interruptions: An example from the flightdeck. *Proceedings of the human factors and ergonomics society annual meeting*, *40* (4), 249–253). Sage CA: Los Angeles, CA: Sage Publications. 10.1177/154193129604000423

[CR27] Lee, B. C., & Duffy, V. G. (2015). The effects of task interruption on human performance: A study of the systematic classification of human behavior and interruption frequency. *Human Factors and Ergonomics in Manufacturing & Service Industries*, *25*(2), 137–152. 10.1002/hfm.20603

[CR29] Monk, C. A. (2004). The effect of frequent versus infrequent interruptions on primary task resumption. Proceedings of the Human Factors and Ergonomics Society Annual Meeting, 48, 295–299. 10.1177/154193120404800304

[CR28] Monk, C. A., Trafton, J. G., & Boehm-Davis, D. A. (2008). The effect of interruption duration and demand on resuming suspended goals. *Journal of Experimental Psychology: Applied*, *14*(4), 299–313. 10.1037/a0014402 19102614 10.1037/a0014402

[CR30] Moretti, L., Koch, I., Steinhauser, M., & Schuch, S. (2024). Stimulus-triggered task conflict affects task-selection errors in task switching – A bayesian multinominal processing tree modelling approach. *Journal of Experimental Psychology: Learning Memory and Cognition*, *50*(2), 230–243. 10.1037/xlm0001245 37155281 10.1037/xlm0001245

[CR31] Peirce, J., Gray, J. R., Simpson, S., MacAskill, M., Höchenberger, R., Sogo, H., & Lindeløv, J. K. (2019). PsychoPy2: Experiments in behavior made easy. *Behavior Research Methods*, *51*(1), 195–203. 10.3758/s13428-018-01193-y 30734206 10.3758/s13428-018-01193-y PMC6420413

[CR32] Piątkowski, K., Beaman, C. P., Jones, D. M., Zawadzka, K., & Hanczakowski, M. (2024). Forgetting during interruptions: the role of goal similarity. *Journal of Cognitive Psychology*, *36*(5), 560–575. 10.1080/20445911.2024.2313570

[CR33] Powers, S. A., & Scerbo, M. W. (2022). Examining the effects of interruption timing and frequency on performance: An update. Proceedings of the Human Factors and Ergonomics Society Annual Meeting, 66(1), 702–706. 10.1177/1071181322661418

[CR34] Raban, M. Z., & Westbrook, J. I. (2014). Are interventions to reduce interruptions and errors during medication administration effective? a systematic review. *BMJ Quality & Safety*, *23*(5), 414–421. 10.1136/bmjqs-2013-002118 10.1136/bmjqs-2013-002118 PMC3995243 23980188

[CR35] Radović, T., & Manzey, D. (2019). The impact of a mnemonic acronym on learning and performing a procedural task and its resilience toward interruptions. *Frontiers in Psychology*, *10*, 493110. 10.3389/fpsyg.2019.02522 10.3389/fpsyg.2019.02522 PMC6851171 31781008

[CR36] Radović, T., & Manzey, D. (2022). Effects of complexity and similarity of an interruption task on resilience toward interruptions in a procedural task with sequential constraints. *Journal of Experimental Psychology: Human Perception and Performance*, *48*(2), 159–173. 10.1037/xhp0000981 35225631 10.1037/xhp0000981

[CR37] Radović, T., Rieger, T., & Manzey, D. (2022). A global and local perspective of interruption frequency in a visual search task. *Frontiers in Psychology*, *13*, 951048. 10.3389/fpsyg.2022.951048 36186383 10.3389/fpsyg.2022.951048 PMC9524370

[CR38] Ratwani, R., Fairbanks, T., Savage, E., Adams, K., Wittie, M., Boone, E., & Gettinger, A. (2016). Mind the gap. *Applied Clinical Informatics*, *7*(4), 1069–1087. 10.4338/ACI-2016-06-R-0105 27847961 10.4338/ACI-2016-06-R-0105 PMC5228144

[CR39] Speier, C., Valacich, J. S., & Vessey, I. (1999). The influence of task interruption on individual decision making: An information overload perspective. *Decision Sciences*, *30*(2), 337–360. 10.1111/j.1540-5915.1999.tb01613.x

[CR40] Strivens, A., Koch, I., & Lavric, A. (2024). Does preparation help to switch auditory attention between simultaneous voices: Effects of switch probability and prevalence of conflict. *Attention Perception & Psychophysics*, *86*(3), 750–767. 10.3758/s13414-023-02841-y 10.3758/s13414-023-02841-y PMC11062987 38212478

[CR41] Westbrook, J. I., Coiera, E., Dunsmuir, W. T. M., Brown, B. M., Kelk, N., Paoloni, R., & Tran, C. (2010a). The impact of interruptions on clinical task completion. *Quality & Safety in Health Care*, *19*(4), 284–289. 10.1136/qshc.2009.039255 20463369 10.1136/qshc.2009.039255

[CR42] Westbrook, J. I., Woods, A., Rob, M. I., Dunsmuir, W. T. M., & Day, R. O. (2010b). Association of interruptions with an increased risk and severity of medication administration errors. *Archives of Internal Medicine*, *170*(8), 683–690. 10.1001/archinternmed.2010.65 20421552 10.1001/archinternmed.2010.65

[CR43] Westbrook, J. I., Li, L., Hooper, T. D., Raban, M. Z., Middleton, S., & Lehnbom, E. C. (2017). Effectiveness of a ‘Do not interrupt’ bundled intervention to reduce interruptions during medication administration: a cluster randomised controlled feasibility study. *BMJ Quality & Safety*, *26*(9), 734–742. 10.1136/bmjqs-2016-006123 10.1136/bmjqs-2016-006123 PMC5574391 28232390

[CR44] Wilson, M. K., Farrell, S., Visser, T. A. W., & Loft, S. (2018). Remembering to execute deferred tasks in simulated air traffic control: The impact of interruptions. *Journal of Experimental Psychology: Applied*, *24*(3), 360–379. 10.1037/xap0000171 30047752 10.1037/xap0000171

[CR45] Zijlstra, F. R. H., Roe, R. A., Leonora, A. B., & Krediet, I. (1999). Temporal factors in mental work: Effects of interrupted activities. *Journal of Occupational and Organizational Psychology*, *72*(2), 163–185. 10.1348/096317999166581

